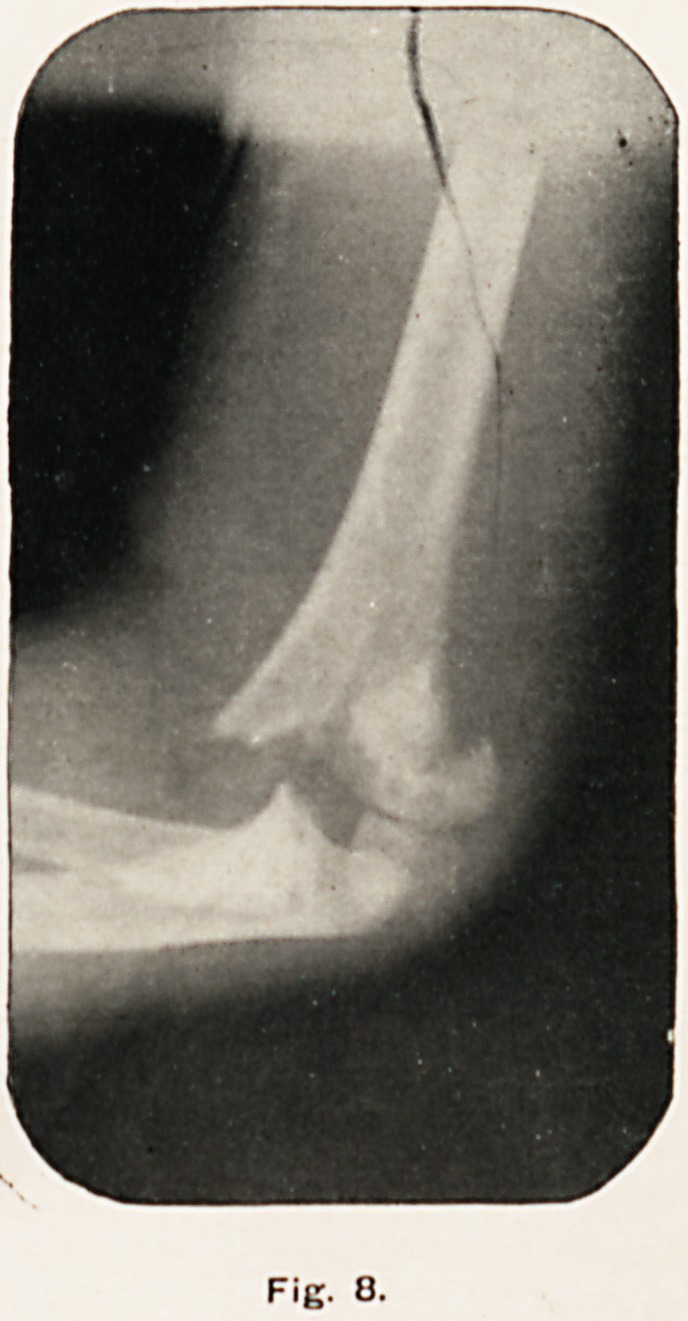# The X-Ray History of a Fracture

**Published:** 1900-09

**Authors:** James Taylor

**Affiliations:** Medical Officer in charge of the X-ray Department of the Bristol Royal Infirmary


### THE X-RAY HISTORY OF A FRACTURE.

BY

James Taylor, M.R.C.S.,

*Medical Officer in charge of the X-ray Department of the Bristol Royal Infirmary.*

On November 20th, 1899, a boy, aged 11 years, whilst at play, received a push from behind and fell on the point of his left elbow. When admitted to the casualty room of the Bristol Royal Infirmary, there was great distortion of the limb, the elbow being carried completely backwards. Fracture of the lower epiphysis of the humerus was diagnosed, and before the reduction of the fracture, the limb was temporarily supported on a straight wooden splint and a skiagram (Fig. 1) was taken. This showed a fracture through the diaphysis of the lower end of the humerus—the epiphysis with the part of the shaft immediately above it, and the condylar ridges of the humerus,

Fig 1.

Fig. 2.

Fig. 3.

Fig. 4.

Fig. 5.

Fig. 6.

Fig. 7.

Fig. 8.

being carried directly backwards. There was no dislocation, the trochlea being in the great sigmoid cavity.

The fracture was easily reduced, and the limb put up on a wooden angular splint, the fracture being apparently in good position. Fig. 2 is the skiagram taken the day after the accident (November 21st). The elbow was very much swollen. Clinically the fracture seemed to be in good position, but the skiagram shows the fragments in anything but apposition. The fractured shaft of the humerus is nearly touching the radius and ulna, and the lower fragment is still much displaced backwards. This being the case, the boy was admitted to the Infirmary under the care of Mr. Bush (to whom I am indebted for permission to publish the case). He was put under an anæsthetic, and the arm was manipulated till the fragments seemed in better position. He was put to bed with a wire angular splint applied to the flexor surface of the arm.

Fig. 3 was taken the day after the operation. It will be seen that the fragments are in much better position, but the end of the fractured shaft of the humerus is still too near the radius and ulna. (The wire splint is seen applied to the arm. Note the incomplete ossification of the trochlea and olecranon.) To remedy this, extension was applied in the following manner: A weight of 10 lbs. was attached to the splint on the forearm and carried over a pulley at the foot of the bed, counter-extension being applied by means of a bandage carried under the axilla and fastened to the head of the bed, the wrist being at the same time supported by a sling round the neck.

Fig. 4 was taken the day after extension was applied. In this it will be seen that the lower fragment is already brought nearly in line with the shaft of the humerus.

Fig. 5 was taken on December 9th, nineteen days after the accident. Callus is distinctly to be seen forming between the fragments.

Fig. 6 was taken on December 28th, five weeks after the accident. There was a large mass of callus on the front of the humerus to be felt at this time, but the only evidence of it in the skiagram is in the spicules of new bone to be seen on the anterior surface of the shaft of the humerus and in the new bone being formed between the fragments. The fragments were now firmly united.

Fig. 7 was taken on July 6th, 1900, nearly eight months after the accident. The fragments have united in good position, as shown by the continuity of the posterior surface of the diaphysis with the epiphysis of the humerus; the sharp end of the fractured shaft anteriorly is apparently in process of being rounded off, the extreme point (where absorption is taking place) being much more transparent than the rest of the bone. The elbow-joint seems quite normal.

The boy states that he "does not notice anything the matter with his arm." He can touch the back of his neck, button his collar, and, with the exception of a slight impairment of flexion, all his movements are practically normal.

This series of pictures affords an example of the use of skiagraphy, not only in the diagnosis of fractures, but also in demonstrating the effect and directing the course of such treatment as may be adopted.

This fracture before the days of skiagraphy would have been diagnosed as separation of the lower epiphysis of the humerus —in fact this diagnosis was made when the boy was first seen —the arm put up on an angular splint, and very likely the patient would have eventually recovered with an elbow he could not have flexed beyond a right angle.

To show how impossible it is to make an exact diagnosis by ordinary clinical methods, Fig. 8 has been introduced. It is the skiagram of a case that, curiously enough, was admitted to the casualty room a day or two after the case just reported. The deformity of the elbow was almost precisely the same as in the first case, but on taking a skiagram it was seen that this was a case of true separation of the epiphysis.

## Figures and Tables

**Fig 1. f1:**
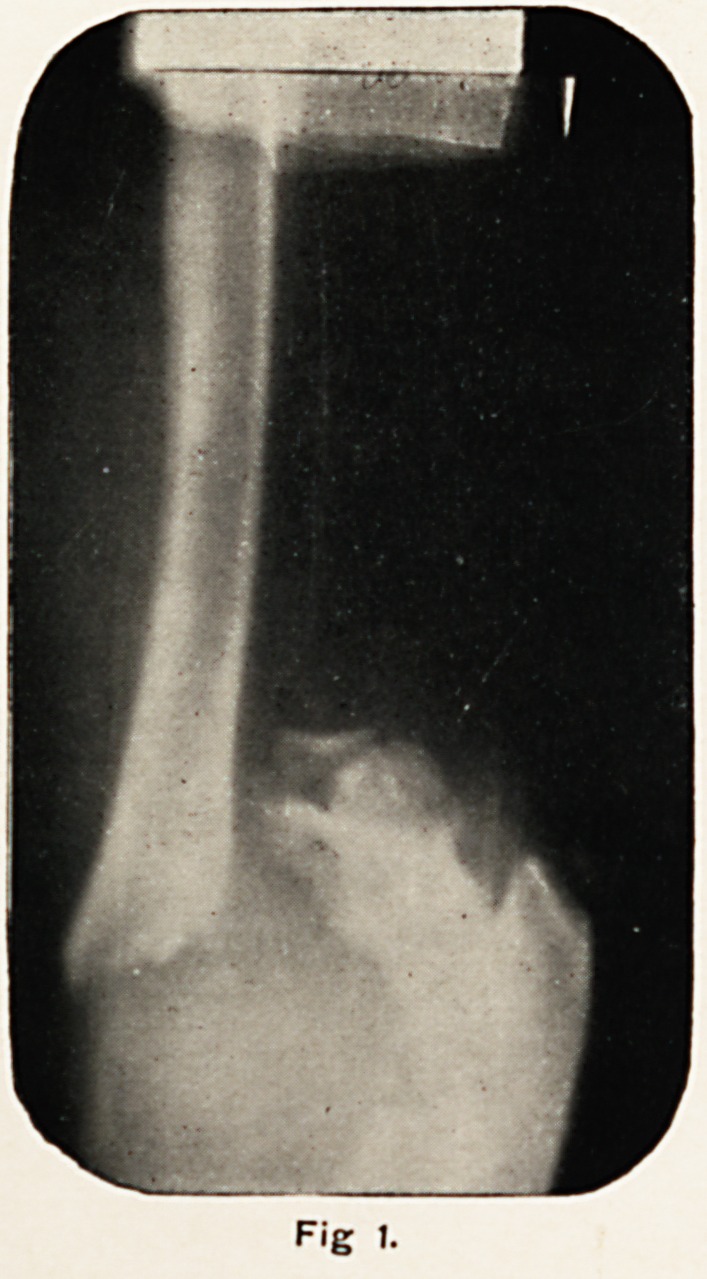


**Fig. 2. f2:**
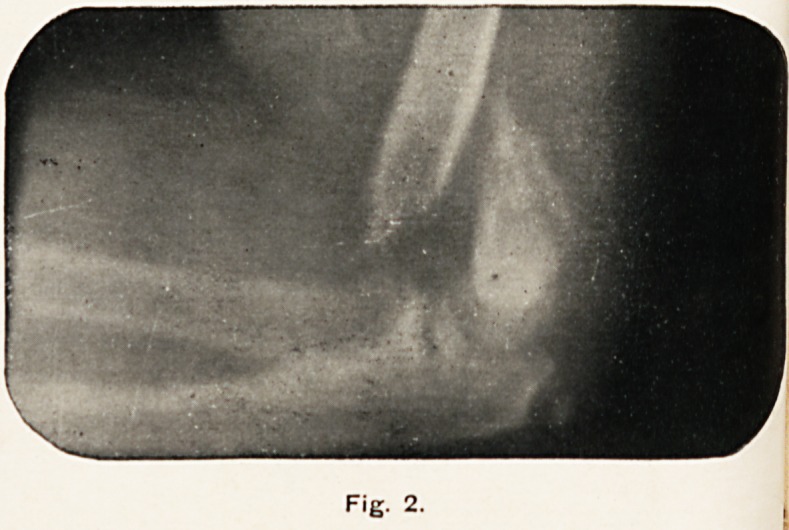


**Fig. 3. f3:**
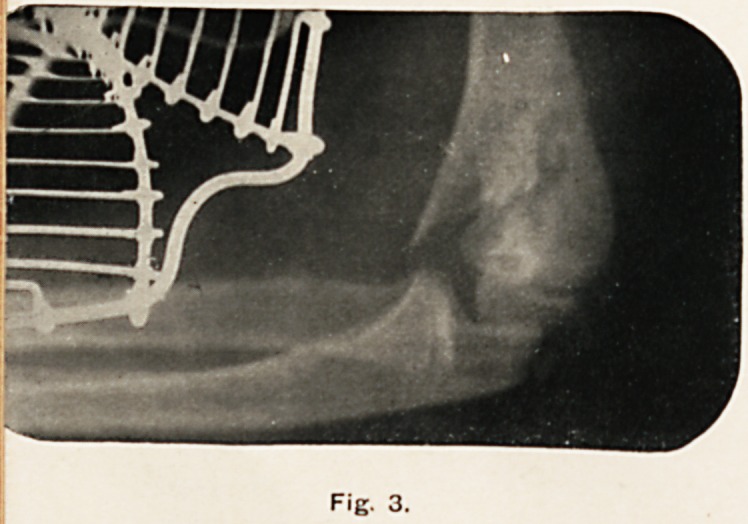


**Fig. 4. f4:**
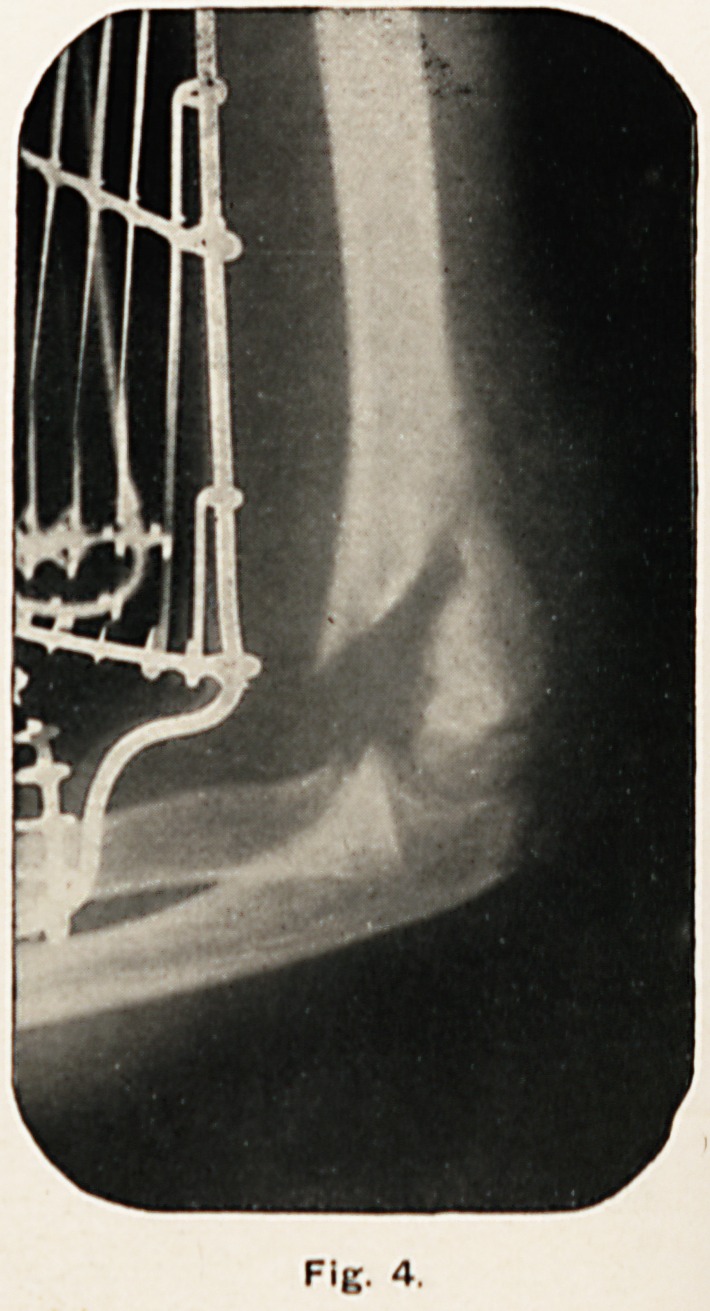


**Fig. 5. f5:**
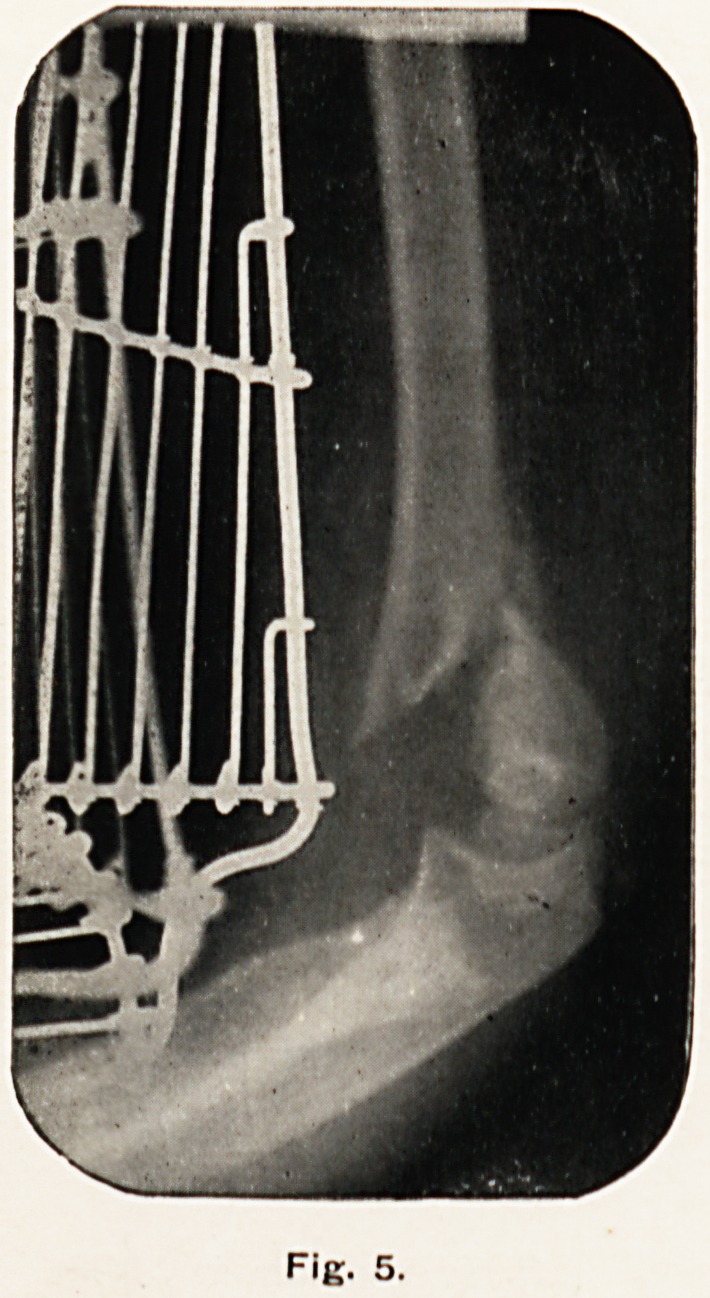


**Fig. 6. f6:**
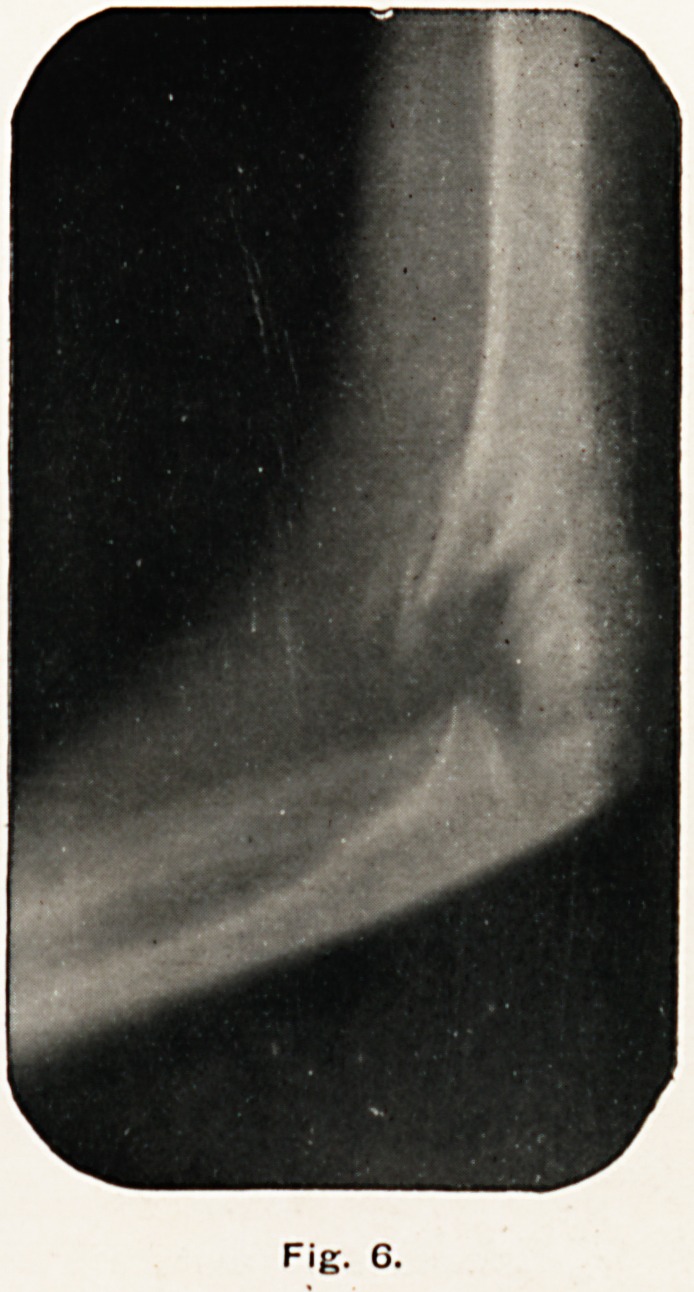


**Fig. 7. f7:**
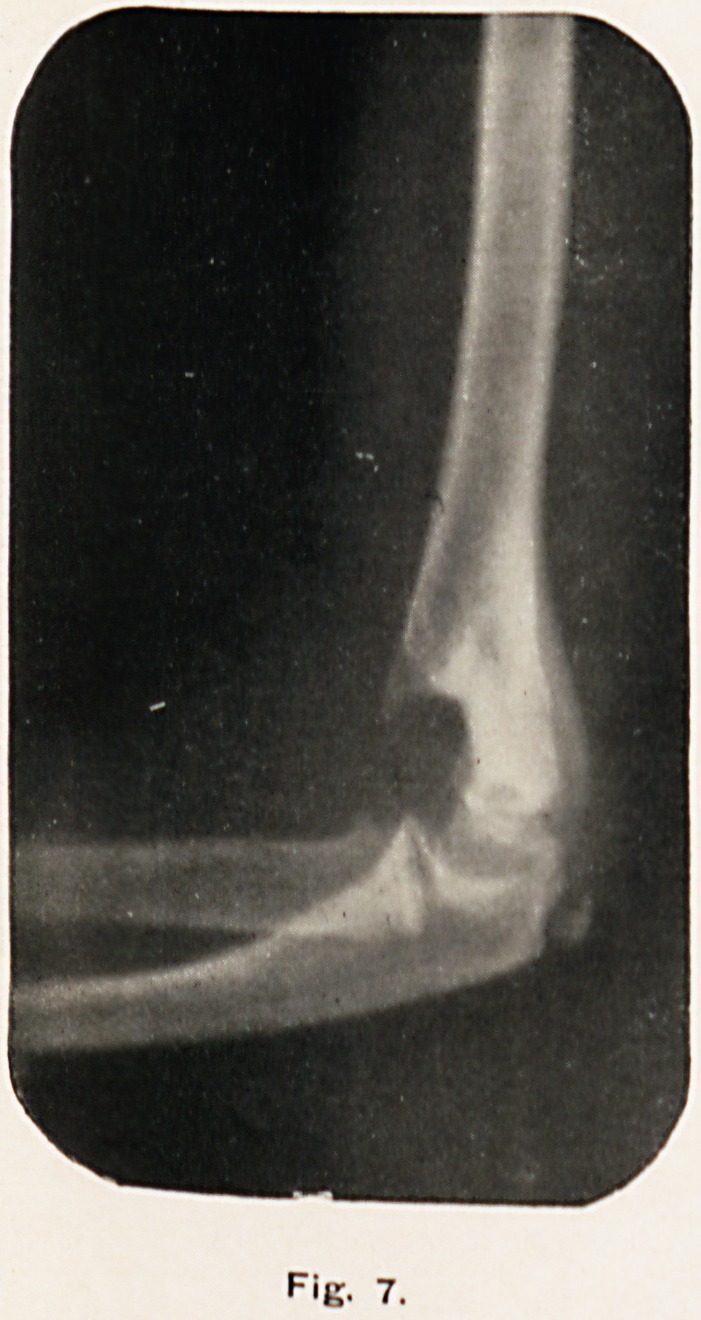


**Fig. 8. f8:**